# Outcomes of palliative care consultation in patients with ESRD who received cardiopulmonary resuscitation 

**DOI:** 10.5414/CN110096

**Published:** 2021-04-09

**Authors:** Caroline M. Hsu, Haris Murad, Kathleen Neuendorf, Tianming Gao, Jesse D. Schold, Fahad Saeed

**Affiliations:** 1 Department of Medicine, Division of Nephrology, Tufts Medical Center, Boston, MA,; 2 Department of Medicine, Division of Nephrology, Washington University of St. Louis, St. Louis, MO,; 3 Department of Quantitative Health Sciences,; 4 Department of Palliative and Supportive Care, Cleveland Clinic, Cleveland, OH and; 5 Department of Medicine, Department of Public Health, Division of Nephrology, Division of Palliative Care, University of Rochester School of Medicine and Dentistry, Rochester, NY, USA

**Keywords:** dialysis, cardiopulmonary arrest, palliative care, end-stage renal disease

## Abstract

Background: The majority of dialysis patients receive aggressive burdensome treatment near the end of life. Currently, we lack interventions to improve end-of-life care (EoLC) for these patients. We examined the association of palliative care consultation with improving EoLC for critically ill patients with end-stage renal disease (ESRD) who received cardiopulmonary resuscitation (CPR). Materials and methods: In this retrospective study, we included patients with ESRD admitted to a large academic center who received CPR either prior to or during their hospital stay. Over 8 years, 17 out of 403 patients received palliative care consultation during their hospital stay; consultations were not standardized in their content. Main outcomes of interest to operationalize better EoLC were: (1) change in code status from full code to do not resuscitate (DNR) and (2) withdrawal from intensive care. Results: Of the patients studied, 60.5% were African-American and 43.2% were female. Demographic differences between those with palliative care consultation and those with usual care were not statistically significant. Palliative care consultation was associated with higher odds of change in code status to DNR (odds ratio 8.10, 95% confidence interval 2.19 – 29.94) and withdrawal from intensive care (odds ratio 8.82, 95% confidence interval 2.69 – 28.91) in patients with ESRD who had received CPR. Palliative care consultation was not associated with any change in in-hospital mortality. Conclusion: Palliative care consultation needs to be considered for hospitalized ESRD patients with limited expected prognoses as it may reduce aggressive and burdensome therapies at the end of life. Furthermore, primary palliative care skills such as communication and decision-making should be taught to nephrologists to improve EoLC for dialysis patients.

## Introduction 

The Institute of Medicine has called for better end-of-life care (EoLC) for patients with serious illnesses such as those receiving maintenance dialysis [1]. Although patients receiving dialysis have a mortality rate and disease burden comparable to many cancers, a higher proportion of these patients still receive aggressive treatment during their final days [2]. Among patients who receive in-hospital cardiopulmonary resuscitation (CPR), patients with end-stage renal disease (ESRD) have higher mortality, shorter median survival, and higher likelihood of discharge to a nursing home [3, 4]. Furthermore, aggressive EoLC not only worsens patients’ quality of life but also portends a higher risk of depression for caregivers [5, 6]. In contrast, the use of comfort-focused treatments near the end of life (EoL) correlates with better patient and caregiver quality of life [5]. Thus, the Renal Physicians Association (RPA), American Society of Nephrology (ASN), and Kidney Disease Improving Global Outcomes foundation (KDIGO) have asserted that patients receiving maintenance dialysis need better EoLC by implementing advance care planning and goals-of-care discussions in both outpatient and inpatient settings [7, 8]. 

Despite these national and international guidelines, advance care planning (ACP) in patients receiving dialysis is not widely completed. Among patients with ESRD, about half have advance directives, of which only a small fraction (~ 3%) address plans for discontinuing dialysis near the EoL [9]. ACP completion rates are not higher for more frail patients; fewer than half of patients with ESRD living in nursing homes have completed any type of advance directive [10]. Thus, the EoL wishes of many of these patients are unknown; therefore in the event of cardiopulmonary arrest, they frequently undergo CPR, experience prolonged hospitalizations, and receive aggressive treatments near the EoL [3, 4, 10, 11]. It is therefore critical to analyze the impact of clinical interventions such as palliative care consultation that have the potential to allow a less aggressive and more comfort-oriented EoL course. 

Palliative care physicians are experts in patient-centered communication, goals of care, and prognostic discussions [12]. Among chronically ill patients, effective patient-physician communication increases the likelihood of receiving care concordant with their goals and values and reduces the likelihood of dying in an intensive care unit (ICU) and undergoing aggressive procedures such as mechanical ventilation and CPR, whose effectiveness is limited in this population [12, 13, 14, 15, 16, 17]. Guided by patients’ preferences and facilitated by high-quality communication skills, such discussions weigh the benefits against the harms of aggressive interventions by forecasting prognosis, thereby enhancing families’ satisfaction with medical care and EoL decision-making [5]. Palliative care often enables patients and families to come to terms with the gravity of the situation, allowing for a death that is not medicalized [18]. However, there remains a paucity of data on outcomes of palliative care consultation in critically ill patients with ESRD with a limited prognosis after they have received CPR. To address the literature gap in this critically ill patient population, we analyzed the outcomes of palliative care consultation operationalized by: (1) a change in code status to do not resuscitate (DNR) after receiving CPR, and (2) withdrawal from intensive care. 

## Materials and methods 

On the basis of an electronic medical records query, we identified all patients 18 years or older at the Cleveland Clinic Foundation hospitals who had received in-hospital CPR or who were admitted after receiving CPR between January 2006 and December 2014. Patients were initially screened by billing codes for CPR (ICD 9 diagnostic codes 99.60 and 99.63), end-stage renal disease (585.6), and dialysis (54.98 for peritoneal dialysis and 39.95 for hemodialysis). CPR and maintenance dialysis status were further confirmed by chart review by the second author (HM). Patients with acute kidney injury were excluded from the study. Additional information was collected by chart review, namely patient characteristics (age, race, sex, duration of dialysis, pre-defined comorbid conditions), CPR characteristics (site of CPR, presenting rhythm), characteristics of the index hospitalization (length of stay, procedures, in-hospital mortality, discharge destination), mortality for the same hospitalization and at the time of chart review for the initial survivors, and outcomes (code status change to DNR, withdrawal from intensive care). The Cleveland Clinic Institutional Review Board approved the study (IRB no. 14-1282). 

Some information was unable to be obtained or completed. Among all participants, data is missing for 3 patients regarding site of CPR, for 40 patients regarding presenting rhythm, for 41 patients regarding need for mechanical ventilation, for 1 patient regarding in-hospital mortality, and for 21 patients regarding discharge destination. 

During the study period, consults to the palliative medicine service were performed by physicians, nurse practitioners, and physician assistants. The consult service had 2 – 5 independent practitioners who made recommendations according to routine clinical practices. 

### Statistical analysis 

We used Studio 1.0.153 to conduct statistical analyses. Descriptive statistics for all the study variables (mean and standard deviation for continuous variables, and frequency and percentage for categorical variables) were examined. Multivariable logistic regression models were fitted to examine the association of palliative care consultation with binary outcomes: (1) change in code status to DNR and (2) withdrawal from intensive care (such as mechanical ventilation, transition to comfort care, and withdrawal from dialysis). Baseline confounders were adjusted in the logistic regression models. 

## Results 

Data was collected from 403 patients on maintenance dialysis who had undergone CPR, and of these, 17 patients had received palliative care consultation. In total, 60.5% of patients were African-American and 43.2% were female. Demographic differences between the two groups (those with palliative care consultation and those with usual care) were not statistically significant (Table 1). 

Duration of dialysis, comorbidity sum, CPR characteristics, and use of invasive procedures did not differ significantly between those with palliative care consultation and those with usual care. 101 patients did not go to the ICU; these were generally patients who either died before reaching an ICU or who survived after receiving brief CPR. 

82.4% of the palliative care group had their code status changed to DNR, as compared to 28.2% in the usual care group. 76.5% of patients in the palliative care group underwent withdrawal from intensive care, as compared to 21.0% in the usual care group. The use of palliative care was not associated with a significant difference in mortality or discharge destination. 

The use of palliative care was associated with a statistically significant difference in code status change to DNR (OR 8.10, CI 2.19 – 29.94, p = 0.002) (Table 2). 

The use of palliative care was also associated with withdrawal from intensive care (OR 8.82, CI 2.69 – 28.91, p = 0.0003) (Table 3). 

Age and location of CPR (in- vs. out-of-hospital) were also associated with the decision to change code status to DNR (Table 2), but not with the decision to withdraw from intensive care (Table 3). 

## Discussion 

In the current sample of critically ill ESRD patients who had undergone CPR, the use of palliative care consultation was associated with less aggressive EoLC, i.e., higher odds of both change in code status to DNR and withdrawal from intensive care. 

Patients with ESRD have palliative care needs similar to other serious illnesses [19, 20]. We have further shown that in ESRD patients who had received CPR, palliative care consultation was associated with change in code status to DNR. A change in code status from full code to DNR signifies an acceptance that an illness is burdensome and may be irreversible, and that it is therefore appropriate to place limits on aggressive care. A frank yet emotionally sensitive discussion about prognosis utilizing palliative care communication skills may lead to a better understanding of the role of CPR by families of critically ill patients [3, 4]. These findings are similar to those published by others in different settings [21, 22, 23, 24, 25, 26]. Despite differences in rates of DNR and withdrawal of intensive care, we did not find a significant difference in mortality between the two groups, which likely reflects the poor outcomes of CPR in dialysis patients. 

The use of palliative care consultation was also associated with higher odds of withdrawal from intensive care. Withdrawal from intensive care is different in the ESRD population as it includes withdrawal from dialysis with a projected estimated survival of a few days to a few weeks [27]. Patients with ESRD are more likely to die in the ICU than patients with dementia or cancer, indicating potentially inadequate advance care planning [11, 28]. Death in an ICU is a surrogate for aggressive therapies before death and has been associated with poorer symptom control and adverse bereavement outcomes [28]. Studies across multiple settings and patient populations have shown an association between palliative care intervention and transition to a comfort-oriented approach, including the use of hospice [20, 29, 30, 31]. Again, this transition signifies a better understanding of prognosis and acceptance of mortality – in the case of our study, without an increase in mortality. Our study was able to confirm previous findings in a unique sample of dialysis patients who had a limited estimated prognosis [3, 4]. 

Our study has several implications. First, palliative care consultation should be obtained more often during hospitalization for ESRD patients following CPR; in our cohort, only 4% received palliative care consultation. Second, we present here a clear association between palliative care and less aggressive treatments at the EoL. Palliative care practitioners are known to use patient-centered communication to open a forum for goals-of-care discussions, especially in the setting of limited prognoses, to ease the transition to a focus on comfort [19]. Our study therefore invites nephrologists and intensivists to learn palliative care-based communication skills [32]. Multiple studies note the association between high-quality communication and less aggressive care [5, 33, 34]. Strong communication involves active and empathic listening, relationship building, and shared decision-making [17, 35]. Workshops of various forms teach these skills and have been shown to improve trainees’ communication skills, whether through patient feedback, simulations, or a set curriculum [36, 37, 38, 39]. Communication skills training should therefore be incorporated into standard nephrology training. Third, our study sample comprises a chronically ill patient population at a time of critical illness. Mandel et al. [40] name several opportunities in the course of CKD/ESRD’s progression to address goals of care, such as dialysis access or transplant referral, recurrent or prolonged hospitalizations, or changes in function or dependence. In this case, cardiopulmonary arrest may be considered a sentinel event to warrant such a discussion. As noted above, studies show a low rate of ACP completion in ESRD patients, suggesting limited access to EoL planning [9, 10, 28]. At the policy level, our study calls for policies to promote goals-of-care discussions and ACP in the ESRD population. Furthermore, it invites nephrology fellowship programs to formally design and implement a primary palliative care curriculum, including training in communication skills for nephrology fellows [32, 41]. 

Our study has several strengths. To our knowledge, this is the first analysis of outcomes of inpatient palliative care provided to post-CPR patients with ESRD. The code status and other data were determined by actual chart review and are not dependent on the accuracy of billing codes. However, our study also has limitations. We recognize that the current study is limited by its retrospective design and other factors including a small sample size potentially causing a selection bias, lack of illness severity score, and non-standardized palliative care consultation. We acknowledge that the derived associations do not imply causality and that the threshold to consult palliative care may vary by clinicians’ attitudes toward palliative care. Lastly, the content of the conversations was not assessed by audiotapes or videos. 

In summary, we found that palliative care consultation was associated with less aggressive treatment at the EoL in post-CPR patients with ESRD. Palliative care consultation should be sought in critically ill ESRD patients after receiving CPR. Interventions to train nephrologists in primary palliative care skills such as EoL communication and decision-making are needed. 

## Prior abstract publication/presentation 

This work was presented at the Mid-Atlantic Nephrology Young Investigators Research Forum as an oral presentation. 

## Funding 

Dr. Saeed is a recipient of the American Society of Nephrology Carl W. Gottschalk Research Scholar Grant and the Renal Research Institute Grant. Dr. Hsu is funded by an NIDDK Institutional NRSA, T32 DK007777. 

## Conflict of interest 

None. 

**Table 1.** Characteristics of all participants.

	Palliative care N = 17	Usual care N = 386	p-value
Baseline characteristics			
Age	68.1 (11.2)	64.1 (13.7)	0.168
Race group:			0.551
African American	12 (70.6%)	232 (60.1%)	
Caucasian	4 (23.5%)	135 (35.0%)	
Other	1 (5.88%)	19 (4.92%)	
Sex:			0.562
Female	9 (52.9%)	165 (42.7%)	
Male	8 (47.1%)	221 (57.3%)	
Duration of dialysis (months)	35.6 (31.1)	50.7 (48.6)	0.073
Number of extrarenal comorbidities	3.18 (1.19)	3.14 (1.35)	0.903
CPR characteristics			
CPR by site:			0.685
In-hospital	12 (70.6%)	240 (62.7%)	
Out-of-hospital	5 (29.4%)	143 (37.3%)	
Presenting rhythm:			0.054
Asystole	1 (6.25%)	75 (21.6%)	
Ventricular arrhythmia	1 (6.25%)	79 (22.8%)	
Pulseless electrical activity	14 (87.5%)	193 (55.6%)	
Characteristics of index hospitalization			
Length of stay (days)	13.8 (11.7)	7.60 (10.0)	0.047
PEG tube:			0.115
No	14 (82.4%)	360 (93.3%)	
Yes	3 (17.6%)	26 (6.74%)	
Ventilation:			1
No	0 (0.00%)	2 (0.58%)	
Yes	17 (100%)	343 (99.4%)	
Tracheostomy:			0.055
No	13 (76.5%)	354 (91.7%)	
Yes	4 (23.5%)	32 (8.29%)	
ICU stay:			0.009
No	0 (0.00%)	101 (26.2%)	
Yes	17 (100%)	285 (73.8%)	
Death in hospital:			1.000
No	6 (35.3%)	127 (33%)	
Yes	11 (64.7%)	258 (67%)	
Discharge to (% of those discharged):			0.537
Home	0 (0.00%)	40 (36.4%)	
Nursing home or rehab	2 (100%)	70 (63.6%)	
Death (total)			1.000
No	3 (17.6%)	67 (17.5%)	
Yes	14 (82.4%)	316 (82.5%)	
Outcomes			
Code status change to DNR:			< 0.001
No	3 (17.6%)	277 (71.8%)	
Yes	14 (82.4%)	109 (28.2%)	
Withdrawal from intensive care:			< 0.001
No	4 (23.5%)	305 (79.0%)	
Yes	13 (76.5%)	81 (21.0%)	

CPR = cardiopulmonary resuscitation; DNR = do not resuscitate.

**Table 2.** Table 2. Multivariable logistic regression for code status change to DNR.

	Odds ratio	Lower 0.95	Upper 0.95	p-value
Use of palliative care services (yes vs. no)	8.10	2.19	29.9	0.002
Age at first CPR (per increase of 10 years)	1.22	1.01	1.48	0.035
Duration of dialysis dependency (> 12 months)	0.96	0.90	1.02	0.20
Comorbidity sum (> 2 extrarenal comorbid conditions)	0.82	0.57	1.19	0.29
Race group (African American vs. Caucasian)	1.07	0.63	1.81	0.26
Race group (Other vs. Caucasian)	2.38	0.83	6.78	
Sex (female vs. male)	1.16	0.72	1.87	0.56
CPR by group (in-hospital vs. out-of-hospital)	1.82	1.10	3.02	0.021
Presenting rhythm (ventricular arrhythmias vs. asystole)	0.70	0.32	1.51	0.072
Presenting rhythm (pulseless electrical activity vs. asystole)	1.43	0.77	2.66	

CPR = cardiopulmonary resuscitation; DNR = do not resuscitate.

**Table 3.** Table 3. Multivariable logistic regression for withdrawal from intensive care.

	Odds ratio	Lower 0.95	Upper 0.95	p-value
Use of palliative care services (yes vs. no)	8.82	2.69	28.9	0.0003
Age at first CPR (per increase of 10 years)	1.14	0.93	1.39	0.22
Duration of dialysis dependency (> 12 months)	0.99	0.92	1.05	0.70
Comorbidity sum (> 2 extrarenal comorbid conditions)	0.93	0.62	1.37	0.70
Race group (African American vs. Caucasian)	0.95	0.54	1.67	0.68
Race group (Other vs. Caucasian)	1.55	0.50	4.75	
Sex (female vs. male)	0.90	0.53	1.51	0.68
CPR by group (in-hospital vs. out-of-hospital)	1.71	0.98	2.97	0.058
Presenting rhythm (ventricular arrhythmias vs. asystole)	0.66	0.28	1.56	0.12
Presenting rhythm (pulseless electrical activity vs. asystole)	1.36	0.70	2.65	

CPR = cardiopulmonary resuscitation.
